# 2D CTAB-MoSe_2_ Nanosheets and 0D MoSe_2_ Quantum Dots: Facile Top-Down Preparations and Their Peroxidase-Like Catalytic Activity for Colorimetric Detection of Hydrogen Peroxide

**DOI:** 10.3390/nano10102045

**Published:** 2020-10-16

**Authors:** Da-Ren Hang, Ya-Qi Pan, Krishna Hari Sharma, Mitch M. C. Chou, Sk Emdadul Islam, Hui-Fen Wu, Chi-Te Liang

**Affiliations:** 1 Department of Materials and Optoelectronic Science, National Sun Yat-sen University, Kaohsiung 80424, Taiwan; zxcasd010379@gmail.com (Y.-Q.P.); krishnaharisharma.27@gmail.com (K.H.S.); mitch@faculty.nsysu.edu.tw (M.M.C.C.); 2 Center of Crystal Research, National Sun Yat-sen University, Kaohsiung 80424, Taiwan; 3 Department of Physics, National Taiwan University, Taipei 10617, Taiwan; sk.emdadul87@gmail.com; 4 Department of Chemistry, National Sun Yat-sen University, Kaohsiung 80424, Taiwan; hwu@faculty.nsysu.edu.tw

**Keywords:** MoSe_2_ quantum dots, peroxidase-like activity, hydrogen peroxide, few-layer MoSe_2_ nanosheets, colorimetric detection

## Abstract

We report the facile and economic preparation of two-dimensional (2D) and 0D MoSe_2_ nanostructures based on systematic and non-toxic top-down strategies. We demonstrate the intrinsic peroxidase-like activity of these MoSe_2_ nanostructures. The catalytic processes begin with facilitated decomposition of H_2_O_2_ by using MoSe_2_ nanostructures as peroxidase mimetics. In turn, a large amount of generated radicals oxidizes 3,3,5,5-tetramethylbenzidine (TMB) to produce a visible color reaction. The enzymatic kinetics of our MoSe_2_ nanostructures complies with typical Michaelis–Menten theory. Catalytic kinetics study reveals a ping–pong mechanism. Moreover, the primary radical responsible for the oxidation of TMB was identified to be Ȯ_2_^−^ by active species-trapping experiments. Based on the peroxidase mimicking property, we developed a new colorimetric method for H_2_O_2_ detection by using 2D and 0D MoSe_2_ nanostructures. It is shown that the colorimetric sensing capability of our MoSe_2_ catalysts is comparable to other 2D materials-based colorimetric platforms. For instance, the linear range of H_2_O_2_ detection is between 10 and 250 μM by using 2D functionalized MoSe_2_ nanosheets as an artificial enzyme. Our work develops a systematic approach to use 2D materials to construct novel enzyme-free mimetic for a visual assay of H_2_O_2_, which has promising prospects in medical diagnosis and food security monitoring.

## 1. Introduction

The development of convenient and sensitive detection of hydrogen peroxide (H_2_O_2_) is in high demand in the fields of food security, environmental monitoring and biochemical analysis. H_2_O_2_, produced from the incomplete reduction of O_2_, can be found as a byproduct in diverse biological processes. Higher amounts than normal of cellular H_2_O_2_ have been linked to the risk of a few diseases including Parkinson’s disease and cancer development [1,2]. Thus, it is of practical importance to analyze and detect H_2_O_2_ by a simple, sensitive and economic method. So far, various techniques for H_2_O_2_ determination have been explored, such as fluorometry [3,4], cellular imaging [5], electrochemistry [6,7], and the colorimetric method [8,9]. Among these approaches, the colorimetric method has drawn a lot of attention due to its convenient operation, visibility, facile miniaturization, and low cost [10,11]. In this respect, natural enzymes were extensively used for the detection of H_2_O_2_ due to its catalysis capability under mild conditions. Nevertheless, these conventional enzymes usually suffer from the disadvantages of low stability against harsh conditions and high expenditures for preparation and purification. Consequently, researchers actively sought artificial enzyme-mimic materials without these shortcomings. Nanomaterials are currently regarded as a rich source to synthesize desired alternative mimic enzymes with the benefits of low cost, plentiful raw materials, and ease in purification and storage. Many nanomaterials with intrinsic enzyme-mimetic activity analogous to that of natural enzymes were fabricated, such as metal organic frameworks [12], Pt nanoclusters [13], silver nanoparticles [14], and gold nanoparticles [15]. Although enormous progress has been made, the discovery and development of novel promising artificial peroxidase mimics is still in urgent need.

With the persistent advancement of nanotechnology and materials science, two-dimensional (2D) nanomaterials beyond graphene have received much attention because of many fascinating chemical and physical properties. The transition metal dichalcogenides (TMDs), a family of layered compound materials consisting of 2D sheets weakly bound by van der Waals interactions, is the most renowned group of emerging 2D materials. They have shown huge promise in a wide range of applications. In particular, TMD nanostructures have shown good potential in biomedical applications due to their large surface area, low cytotoxicity, and higher structural rigidity than other 2D nanomaterials. For instance, it was found that TMDs exhibited lower cytotoxicity than typical graphene and its analogues [16]. As for structural rigidity, commonly used graphene and hexagonal boron-nitride have relatively low flexural rigidity around 3.5 eV Å^2^/atom. On the other hand, these values for MoS_2_ and WS_2_ are 27 eV Å^2^/atom and 30 eV Å^2^/atom, respectively [17]. These properties should make 2D TMDs appropriate for biomedical applications. Finally, TMDs can remain stable in liquid due to the lack of dangling bonds on the surface, which supports their use in biomedical applications.

Molybdenum disulfide (MoS_2_), the most prominent member of the TMD family, possesses distinctive properties and has found diverse successful applications in electronics [18], energy devices [19], photocatalysis [20], and sensors [21,22]. In particular, the peroxidase-like catalytic ability of a few MoS_2_ nanostructures has been shown by researchers [23,24]. While a large amount of investigations is devoted to MoS_2_, considerably less attention has been devoted to molybdenum diselenide (MoSe_2_) [25]. 2H-MoSe_2_, also with a graphene-like lamellar structure, is a semiconductor whose bandgap energy increases from ~1.1 eV in bulk to ~1.55 eV in ultrathin form with atomic thickness. In a previous comparative study, Gholamvand and coworkers concluded that MoSe_2_ is the most effective electrocatalyst among TMDs [26]. Recent studies also showed that few-layered MoSe_2_ nanosheets (NSs) could be a promising candidate with peroxidase-like activity and good biocompatibility [27,28]. Moreover, inspired by the fact that selenium-containing enzymes are generally prevalent in the biosphere and their active sites usually involve selenium, we thus turned our attention to the investigation of nanoscale MoSe_2_ in this respect. Even though MoSe_2_ is expected to function as efficient peroxidase mimetics for colorimetric detection, so far little progress has been made in this respect. Its extended topics, such as surface modification and variation in dimensionality, were rarely studied for use in colorimetric detection. Here, we intend to fill the gap along this exploration. For instance, TMD in quantum dot (QD) form deserves more investigation because their pronounced quantum confinement effects (QCE) and edge effects further aid applications in catalysts and sensing [29,30]. Significant enhancement in photoluminescence (PL) quantum efficiency by QCE is favorable to develop a sensor by optical means [31,32]. Moreover, a few optical properties in the strong coupling regime for semiconductor QDs and nanostructures could be implemented to strengthen the functionality of biosensors [33,34,35].

Liquid-phase synthesis routes are suitable to produce TMD nanostructures in large quantity at low cost. In general, solution-based synthesis approaches can be divided into “top-down” methods and “bottom-up” methods. For bottom-up wet-chemical synthesis methods, specific precursors are needed and a high-temperature and high-pressure environment is required. Among top-down approaches, liquid phase exfoliation (LPE) is a powerful technique to efficiently exfoliate various types of layered crystals into few-layer nanosheets or even QDs [36,37]. The basic protocol of LPE technique is very general and only parent crystal is needed instead of the need for specific precursors in bottom-up chemical methods.

In this paper, we prepared two types of low-dimensional MoSe_2_ nanostructures based on top-down techniques. In the first case, LPE-derived 2D MoSe_2_ nanosheets were functionalized with cetyltrimethyl ammonium bromide (CTAB). It is expected that the CTAB surfactant could aid exfoliation efficiency and prevent 2D nanosheets from restacking or agglomeration [38]. Secondly, 0D MoSe_2_ QDs were obtained based on top-down exfoliation approaches. For usual 0D TMD QDs derived from LPE, longer ultrasonication time and higher power were typically adopted, which could easily deform the microstructure and result in higher density of surface traps states. As surface-to-volume ratio is rather large for QDs, these deep trap states pose negative impact to many applications. In our work, a novel and efficient ultrasonication-assisted solvothermal exfoliation technique is firstly introduced for preparing small size and high-quality MoSe_2_ QDs. In the initial probe-assistant ultrasonication exfoliation phase, MoSe_2_ bulk is broken into nanosheets or nanoparticles by the acoustic cavitation effect. The sonication time is kept short in this stage. Next, the solvothermal treatment with a polar solvent continuously weakens the van der Waals forces of thinned MoSe_2_ and break it up into small 0D QDs. We show that our CTAB-modified MoSe_2_ NSs and 0D MoSe_2_ QDs are able to efficiently catalyze the oxidation of 3,3,5,5-tetramethylbenzidine (TMB) in the presence of H_2_O_2_ to produce a colored product. On this basis, we have successfully demonstrated novel platforms for colorimetric detection of H_2_O_2_. It is found that the sensing capability of our MoSe_2_ systems is comparable to those of published 2D materials-based platforms. As far as we know, it is the first time the potential of CTAB-functionalized MoSe_2_ nanosheets and 0D MoSe_2_ QDs have been explored for colorimetric detection of H_2_O_2_. Toxic and high boiling point solvents were not used in our synthesis methods thus our protocols also provide a non-toxic and systematic way to fabricate new 2D nanomaterials for construction of novel colorimetric sensors and for use in extended applications.

## 2. Materials and Methods

### 2.1. Materials and Reagents

MoSe_2_ powder (99.9%), 3,3,5,5-tetramethylbenzidine (TMB), and isopropanol were purchased from Sigma-Aldrich (St. Louis, MO, USA). Cetyltrimethyl ammonium bromide (CTAB) was purchased from Millipore (Burlington, MA, USA). Acetic acid, sodium acetate anhydrous, hydrochloric acid, and hydrogen peroxide (35%) were obtained from Alfa Aesar (Tewksbury, MA, USA). These chemicals were of analytical purity and were used as received. Deionized water (DI water) was used as a solvent throughout.

### 2.2. Methods

#### 2.2.1. Preparation of Surfactant Modified Two-Dimensional (2D) MoSe_2_ Nanosheets (NSs)

**Figure 1:**
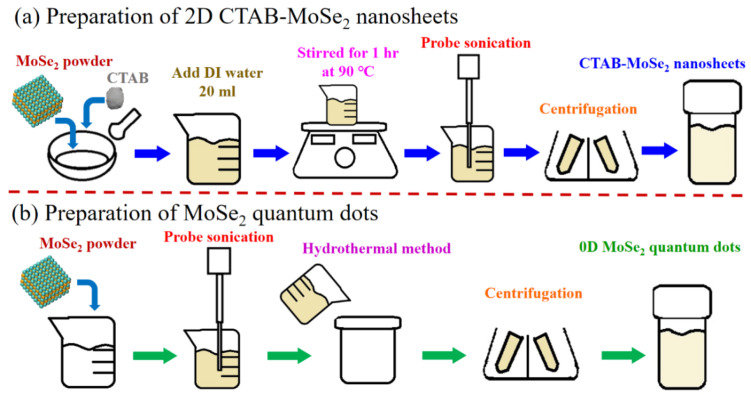
The schematic illustrations for the preparations of (**a**) two-dimensional (2D) cetyltrimethyl ammonium bromide (CTAB)-MoSe_2_ nanosheets and (**b**) 0D MoSe_2_ quantum dots (QDs).

The few-layer CTAB-MoSe_2_ NSs synthesis protocol is based on the grinding-assisted liquid phase exfoliation approach [37]. The synthesis protocol of 2D CTAB-MoSe_2_ NSs is illustrated in Figure 1a. First, 100 mg of MoSe_2_ powder and 50 mg of CTAB were ground for 30 min. The mixture was subsequently dispersed in 20 mL of DI water and stirred for 1 h at 90 °C in a beaker. The solution was probe sonicated for 3 h with a horn sonic tip (Qsonica CL-334) at a power output of 125 W in a water-cooled bath at 20 °C. Residual sediment and thick flakes were further removed by centrifugation at 4000 rpm for 20 min. The upper portion of the supernatant was taken for the next centrifugation for 20 min at the speed of 9000 rpm. The upper portion of the resultant supernatant was transferred to a refrigerator at 4 °C for storage. Finally, the 2D CTAB-MoSe_2_ NS product was collected by another centrifugation at the speed of 9000 rpm.

#### 2.2.2. Preparation of 0D MoSe_2_ QDs

The 0D MoSe_2_ QDs were obtained according to the ultrasonication-assisted solvothermal exfoliation technique. Figure 1b depicts the synthesis procedure of 0D MoSe_2_ QDs. Typically, 100 mg of MoSe_2_ powder was dispersed in 60 mL of 50 vol% **Isopropyl alcohol** (IPA)/DI water mixture in a beaker. Then, the solution was probe sonicated for 1 h with a horn sonic tip (Qsonica CL-334) at a power output of 150 W in a water-cooled bath at 20 °C. Afterward, the resultant dispersions was further transferred to a 60 mL Teflon-lined autoclave and reacted at 200 °C for 24 h. After the autoclave cooled naturally, the supernatant containing MoSe_2_ QDs was centrifuged for 30 min at the speed of 9000 rpm. After that, the upper portion of the supernatant was collected for second centrifugation for 15 min with the same rotation speed. Finally, the MoSe_2_ QD product was collected and then stored in a refrigerator at 4 °C for use.

### 2.3. Characterization

Transmission electron microscopy (TEM) images and high-resolution TEM (HRTEM) images were taken by using a JEOL-3010 transmission electron microscope at an accelerating voltage of 200 kV (Tokyo, Japan). The elemental composition and bonding configuration analysis were carried out by an ultrahigh vacuum JEOL JPS-9010 X-ray photoelectron spectrometer (XPS) equipped with a multi-channel detector. The detected binding energies were calibrated to the C1 s peak at 284.8 eV of the surface adventitious carbon. The ultraviolet–visible (UV–vis) spectra were recorded with a Jasco V-730 spectrophotometer (USA) with a standard 10-mm path length quartz cuvette (Easton, MD, USA). The photoluminescence spectra were measured using a Hitachi F-4500 florescence spectrophotometer connected to a 150 W Xenon lamp as the excitation source. The Raman spectra were recorded in ambient conditions using a confocal microscope linked to a Horiba iHR320 spectrometer (Piscataway, NJ, USA) [39].

### 2.4. Peroxidase-Mimetic Activity of MoSe_2_ Quantum Dots (QDs) and 2D Cetyltrimethyl Ammonium Bromide (CTAB)-MoSe_2_ NSs

To evaluate the catalytic peroxidase-like properties, a blue product was generated by the peroxidase substrate TMB in the presence of H_2_O_2_. In a typical experiment, 600 μL 0.1 mg/mL MoSe_2_ catalyst was incubated with 500 μL acetate buffer solution (0.1 M, pH 3.6), 200 μL H_2_O_2_ (10 mM), 200 μL TMB (5 mM) and 60 μL H_2_O at room temperature for 15 min. Then, the absorbance of the mixture was measured by a Jasco V-730 UV-visible spectrophotometer (Easton, MD, USA). For H_2_O_2_ detection, different contents of H_2_O_2_ were incubated with 600 μL 0.1 mg/mL MoSe_2_ catalyst, 500 μL acetate buffer solution (0.1 M, pH 3.6) and 200 μL TMB (5 mM) at room temperature for 15 min, and then the absorbance at 652 nm was recorded.

## 3. Results and Discussion

### 3.1. Structural Studies

**Figure 2:**
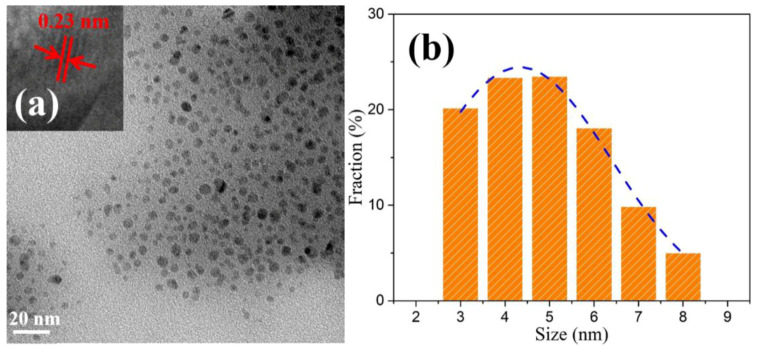
(**a**) Transmission electron microscope (TEM) image of synthesized MoSe_2_ QDs. The inset shows representative high-resolution TEM (HRTEM) image of the MoSe_2_ QD. (**b**) Statistical analysis of the size of MoSe_2_ QDs measured by TEM and its Gaussian fitting curve.

The microstructure of the resultant MoSe_2_ nanomaterials is characterized by transmission electron microscopy (TEM). As shown in Figure 2a, the as-obtained MoSe_2_ QDs reveal a spherical shape without noticeable aggregation, indicating the successful formation of highly dispersive QDs. The statistical analysis of particle size distribution was conducted by counting 700 QD profiles measured by TEM. The outcome is displayed by the histogram in Figure 2b along with its calculated Gaussian fitting curve. The average size of the 0D QDs was determined to be 4.5 nm and up to 80% QDs have their diameters in the narrow range from 3 to 6 nm. A high-resolution TEM (HRTEM) image of a single MoSe_2_ QD in the inset of Figure 2a reveals that the lattice spacing of the synthesized QD was 0.23 nm, which coincides with the (103) plane of hexagonal MoSe_2_ [40].

**Figure 3:**
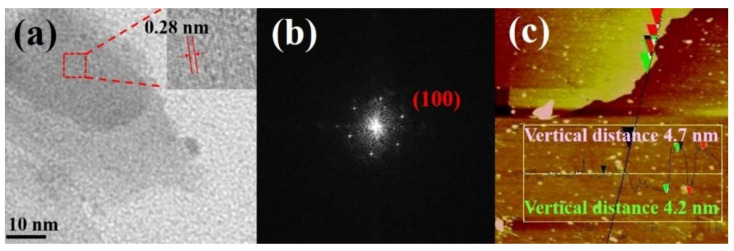
(**a**) TEM image of 2D CTAB-MoSe_2_ prepared by liquid phase exfoliation (LPE). The inset shows its HRTEM image (**b**) The selected area electron diffraction pattern (SAED). (**c**) The atomic force microscopy image of as-obtained 2D CTAB-MoSe_2_ and the corresponding height profile along the black line in the image.

Next, Figure 3a shows the representative TEM image of the as-prepared 2D CTAB-MoSe_2_ nanosheet, in which a sheet-like structure can be found. The HRTEM image in the inset of Figure 3a resolves lattice fringes with lattice spacing of 0.28 nm, which is in agreement with the (100) plane of 2H-MoSe_2_. As shown in Figure 3b, the selected-area electron diffraction (SAED) pattern again verifies the diffraction pattern from the 2H-MoSe_2_ crystal and demonstrates the good crystallinity of the exfoliated nanosheets. Atomic force microscopy (AFM) measurement was adopted to further confirm the 2D nature of CTAB-MoSe_2_ nanosheets. A representative AFM image is shown in Figure 3c and the height profile along the black line was measured. It was found that the nanosheet thickness ranges from 4.2 to 4.7 nm, which confirms the 2D few-layered structure.

### 3.2. Surface Elemental and Valence State Analysis

**Figure 4:**
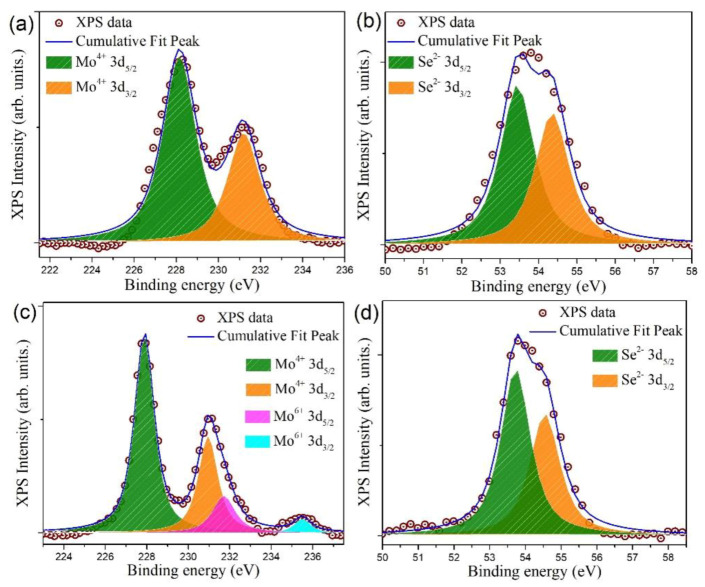
High-resolution X-ray photoelectron spectrometer (XPS) spectra showing the binding energy of (**a**) Mo 3d and (**b**) Se 3d electrons recorded on bulk MoSe_2_ powder. High-resolution core level spectra corresponding to (**c**) Mo 3d and (**d**) Se 3d electrons for MoSe_2_ QDs.

To further shed light on the surface chemical components and oxidation states of our solvothermal-treated MoSe_2_ QDs, XPS was performed on both pristine bulk MoSe_2_ powder and MoSe_2_ QDs. Figure 4a,b show the high-resolution Mo 3d and Se 3d XPS spectra of pristine MoSe_2_ powder, respectively. The two peaks located at 228.1 eV and 231.2 eV correspond to the Mo 3d_5/2_ and Mo 3d_3/2_ peaks of the Mo^4+^ state in MoSe_2_, which is in agreement with previous reports [19,41]. Meanwhile, the peak of Se 3d spectrum can be deconvoluted into two components: the binding energy peaks at 53.4 eV and 54.2 eV are characteristic signals of Se^2−^ 3d_5/2_ and Se^2−^ 3d_3/2_, respectively [42]. In this case, a signal from the Mo^6+^ state was not observed, indicating that there is no noticeable oxidation in our pristine material. Next, the high-resolution Mo 3d and Se 3d spectra of MoSe_2_ QDs are presented in Figure 4c,d, respectively. The deconvolution of Mo 3d spectral region reveals four contributions. The two intense peaks at 227.9 and 231.1 eV belong to the characteristic signals from Mo^4+^ 3d_5/2_ and Mo^4+^ 3d_3/2_, respectively. Furthermore, the other two minor peaks at binding energies of 232.2 and 235.5 eV are ascribed to the Mo(VI) state [37]. This suggests that a small portion of surface Mo^4+^ was oxidized into Mo^6+^ during the imposed reaction [43,44]. As shown in Figure 4d, the two deconvoluted components of the Se 3d doublet appear at 53.5 eV and 54.3 eV, which can be assigned to the Se 3d_5/2_ and Se 3d_3/2_ orbitals of divalent selenide ions (Se^2−^), respectively. It can be confirmed that the binding energies of Mo^4+^ and Se^2-^ orbitals of our prepared QDs do not deviate noticeably from those of the starting material. It is reasonable as no heterostructure formation or other interaction is involved.

### 3.3. Optical Property Studies

**Figure 5:**
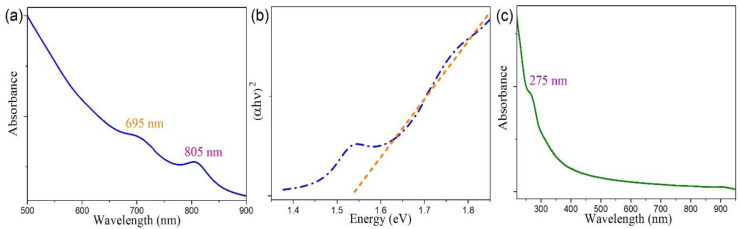
(**a**) Ultraviolet–visible (UV–vis) absorption spectrum of 2D CTAB-MoSe_2_ nanosheets clearly reveals characteristic excitonic structures. (**b**) The corresponding plot versus (*ahv*)^2^ versus *hν* for absorption of 2D CTAB-MoSe_2_, in which the optical band gap energy can be estimated. (**c**) UV–vis absorption spectrum of MoSe_2_ QDs. Note the quenching of previous excitonic features in the spectrum.

It is known that the dimensionality strongly affects the optical properties of nanoscale semiconductors. The optical absorption spectrum of the resultant CTAB-MoSe_2_ nanosheets in dispersion is displayed in Figure 5a. The evident absorption peaks at 805 and 695 nm can be easily identified and they are attributed to the characteristic resonances of A and B excitons, respectively [45,46,47]. Their origin is derived from the transitions between the spin-orbit split valence bands and the lowest conduction band at K and K’ points of the Brillouin zone. Moreover, it is worth of noting that the determined energy separation of 244 meV between the A and B excitonic states is consistent with a previous study on the energy splitting of the exciton states in ultrathin MoSe_2_ nanosheets [48]. Therefore, it provides a quantitative proof of pronounced quantum confinement effect in our exfoliated 2D MoSe_2_ nanosheets. The facile and conventional way to address the optical band gap is by means of the Tauc plot. The absorption coefficient α of a direct band-gap semiconductor can be related to photon energy *hυ* by (α*hυ*)^2^ = *A* (*hυ* − *E*_g_), where *A* is a constant and *E*_g_ is the optical band gap. Figure 5b plots the relationship of (α*hυ*)^2^ versus *hυ*, which demonstrates a linear dependence. The calculated optical gap for the apparent absorption is 1.54 eV (805 nm), which exactly coincides with the A excitonic states and highlights the 2D nature of CTAB-MoSe_2_ nanosheets.

The optical absorption spectrum of the as-prepared 0D MoSe_2_ QDs is in sharp contrast with their 2D counterpart, as shown in Figure 5c. Here, the A and B excitonic features in the absorption completely disappeared. Instead, the absorption feature comprised two absorption bands. The prominent band is centered at around 275 nm, which is ascribed to the intrinsic excitonic absorption of the QDs [49,50]. Such a significant blue-shift of the excitonic features directly reflects the dominating quantum confinement effect and is in accordance with previous studies on other TMD QDs [32,51]. Furthermore, there exists another mild absorption band at longer wavelengths. It is wide and centered about 325 nm with a tail extending to ~400 nm, which will be commented upon later.

**Figure 6:**
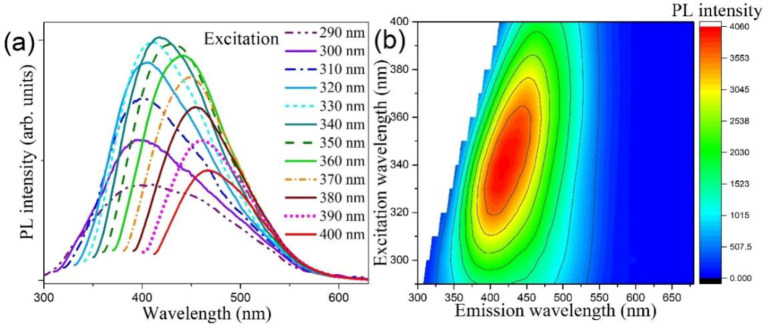
(**a**) Excitation-wavelength dependent photoluminescence (PL) spectra of colloidal MoSe_2_ QDs at room temperature. (**b**) The 2D contour map acquired from the PL spectra. The characteristic contour is due to the pronounced quantum confinement effect.

Photoluminescence (PL) spectroscopy provides a complimentary optical means to probe the electronic structure of semiconductor materials [52,53]. The distinct optical property of TMD QDs is well-suited to be further evidenced by the PL technique. It is easily found that our MoSe_2_ QDs dispersed in aqueous solution emit strong blue fluorescence under irradiation with a typical UV lamp. It is due to the weak interlayer coupling and enhanced quantum efficiency of MoSe_2_ QDs, which is another signature of TMD QDs [54]. To gain a comprehensive view of the emission property, the PL spectra of resultant MoSe_2_ QD dispersion were further taken with different excitation wavelengths, as shown in Figure 6a. It is observed that when the excitation wavelength was increased from 290 to 400 nm, the PL peak position monotonically increased from 390 to 470 nm. Similar excitation-dependent PL behavior has been reported in a few TMD QD reports [32]. In a strong quantum confinement regime, photons with higher energies resonantly excites smaller QDs with wider band gaps, pushing the emission peak to shorter wavelengths. Accordingly, the characteristic excitation-dependent PL behavior derives from the polydispersity of the synthesized QDs. This idiosyncratic variation of PL intensity in response to varying excitation wavelengths can be clearly presented by the 2D color-converted PL contour map as depicted in Figure 6b. We found the strongest emission peaked at 418 nm under an excitation wavelength of 340 nm. This specific wavelength falls coincidentally within the observed absorption band around 325 nm. Thus the close correspondence between the absorption and the emission of the synthesized QDs can be revealed by our optical characterizations.

**Figure 7:**
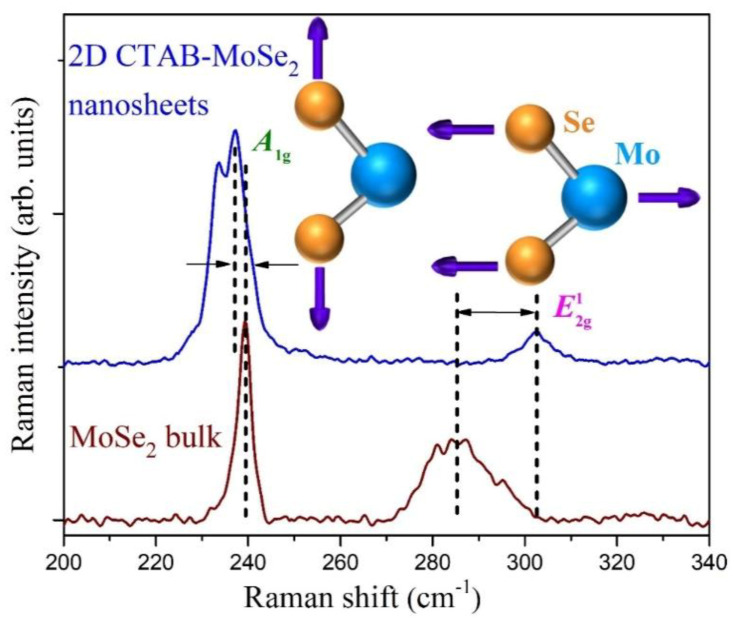
Raman spectra of the source MoSe_2_ bulk powder (brown line) and as-produced 2D CTAB-MoSe_2_ nanosheets (blue line). The Raman shifts are denoted by the dashed lines. The inset sketch depicts the atomic displacements of the two vibrational modes leading to the primary Raman peaks.

Raman spectroscopy was adopted to acquire additional insight into the optical characteristics of 2D CTAB-MoSe_2_ nanosheets. In general, group theory analysis permits bulk TMDs to have four Raman-active modes. However, only two modes are accessible in typical experimental configuration, namely, out-of-plane A_1g_ and in-plane E^1^_2g_ modes. The inset sketch in Figure 7 depicts these two principal Raman-active vibration modes of MoSe_2_. Figure 7 compares the Raman spectra of both MoSe_2_ bulk and 2D CTAB-MoSe_2_ nanosheets. For MoSe_2_ bulk, the A_1g_ mode is located at 239.4 cm^−1^ while the in-plane E^1^_2g_ mode appears at ≈285.5 cm^−1^, which match nicely with literature values [55]. The A_1g_ mode for CTAB-MoSe_2_ nanosheets is red-shifted to 237 cm^−1^, which is attributed to the softening of the vibrational mode [56]. The reduced inter-planar restoring force is another proof for the 2D few-layer structure. In addition, a new peak emerges on the lower-frequency side of the A_1g_ peak. It is ascribed to Davydov splitting of the A_1g_ mode that is accompanied by the suppressed interlayer interaction as reported in TMD nanosheets [57,58]. In contrast, the E^1^_2g_ mode for CTAB-MoSe_2_ shifts to 302 cm^−1^. The dielectric screening of the long-range Coulomb interaction and the surface effects of TMD materials were proposed to be responsible for this blue-shift [59]. The increased energy splitting between the two allowed Raman peaks is in accord with the 2D nature of our CTAB-MoSe_2_ [60,61].

### 3.4. Peroxidase-Like Activities and Steady-State Kinetic Assay

**Figure 8:**
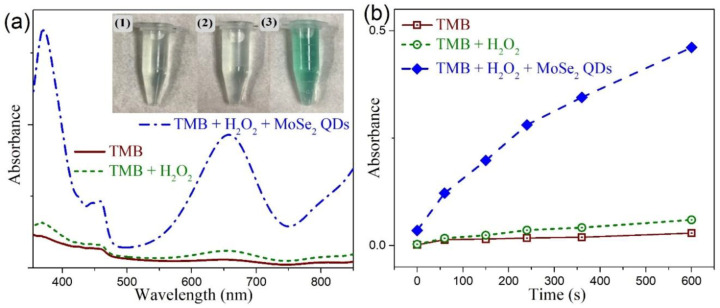
(**a**) UV-visible absorption spectra of (1) 3,3,5,5-tetramethylbenzidine (TMB) solution (brown); (2) TMB–H_2_O_2_ system (green); (3) TMB–H_2_O_2_–MoSe_2_ QDs system (blue). Inset: the corresponding photographs of these reaction systems. (**b**) The time-dependent absorbance changes at 652 nm of these systems.

We evaluated the peroxidase-like activity of MoSe_2_ QDs by using the catalytic oxidation of TMB in the presence of H_2_O_2_, as shown in Figure 8a. The absorption spectrum of the TMB solution showed it is colorless. When only H_2_O_2_ was incubated with TMB, the TMB–H_2_O_2_ systems showed rather weak absorbance at 652 nm. Yet as TMB coexisted with H_2_O_2_ and the MoSe_2_ QDs, a prominent absorption peak of the oxidation products of TMB at 652 nm was observed. Moreover, the color contrast of these system is presented in the inset of Figure 8a. It can be seen that the bare TMB and the TMB–H_2_O_2_ systems are virtually colorless to the naked eye, while TMB–H_2_O_2_–MoSe_2_ QDs system showed an apparent color variation. Figure 8b display time-dependent absorbance changes at 652 nm of these systems. It clearly shows the absorbance at 652 nm increased as the time increased for TMB–H_2_O_2_–MoSe_2_ QDs system. This means that the prepared MoSe_2_ QDs possess the peroxidase-like catalysis capability, which effectively catalyze the oxidation of TMB by H_2_O_2_. On the contrary, rather insignificant and slow oxidation of TMB by the presence of H_2_O_2_ was found for the reference TMB–H_2_O_2_ system. Our results thus demonstrated that the MoSe_2_ QDs can facilitate the oxidation of TMB to oxTMB in the presence of H_2_O_2_ to generate observable color changes. An identical result was also found for our 2D CTAB-MoSe_2_ nanosheets (not shown here).

**Figure 9:**
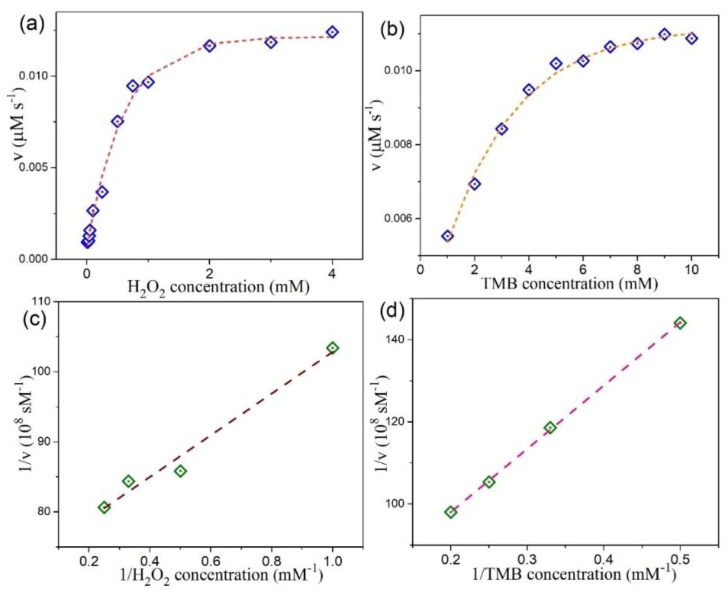
Steady-state kinetic analysis for MoSe_2_ QDs. The reaction velocity (v) was measured when (**a**) the H_2_O_2_ concentration was varied while the concentration of TMB was 5 mM and (**b**) the TMB concentration was varied while the concentration of H_2_O_2_ was 0.75 mM. The corresponding double-reciprocal plots with a fixed concentration of one substrate relative to varying the concentration of the other substrate are displayed in (**c**,**d**).

**Figure 10:**
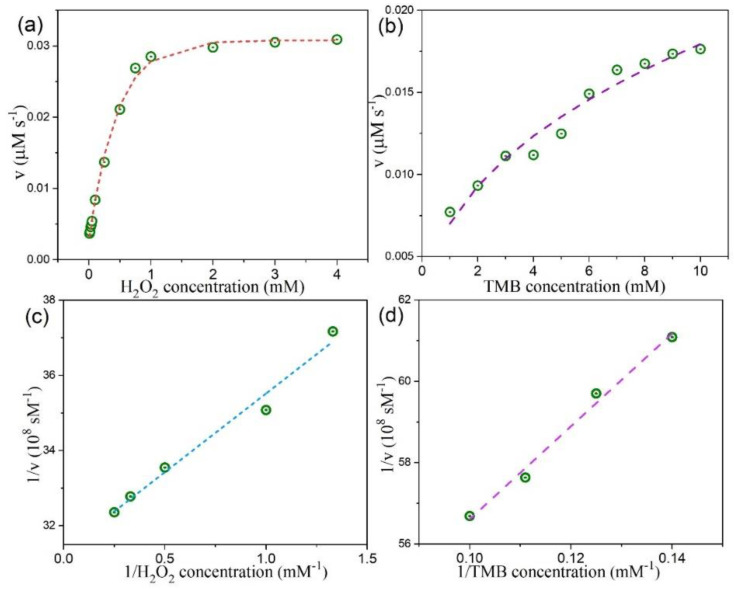
Steady-state kinetic assay of 2D CTAB-MoSe_2_ nanosheets. (**a**) Varying the concentrations of H_2_O_2_ while the concentration of TMB was 5 mM. (**b**) Varying the concentrations of TMB while the concentration of H_2_O_2_ was 0.75 mM. The double-reciprocal plots for the concentration of (**c**) H_2_O_2_ and (**d**) TMB.

The kinetic parameters of the peroxidase-like reaction were harvested by employing the steady-state kinetics analysis. With H_2_O_2_ and TMB as substrates, the measurements were carried out by changing the concentration of one substrate while keeping the other substrate concentration constant. This generates the typical Michaelis–Menten curves, as shown in Figure 9a,b for our MoSe_2_ QDs. For 2D CTAB-MoSe_2_ nanosheets, these curves are plotted in Figure 10a,b. The relevant kinetic parameters like the Michaelis–Menten constant (*K*_m_) and the maximal reaction velocity (*V*_max_) can be extracted from the Lineweaver–Burk plot according to the relation: 1/*v* = (*K*_m_/*V*_max_) × (1/[*S*]) + 1/*V*_max_, where *v* stands for the initial velocity and [*S*] signifies the concentration of the substrate [62,63]. Figure 9c,d display the L-B plot for our 0D MoSe_2_ QDs while Figure 10c,d illustrate the L-B plot for 2D CTAB-MoSe_2_ nanosheets. The calculated results are listed in Table S1. The *K*_m_ value is regarded as an important index that measures the binding affinity of enzyme to the substrates. A smaller value of *K*_m_ usually indicates a higher affinity between the enzyme and the substrate. It is found that the *K_m_* value of 2D CTAB-MoSe_2_ for H_2_O_2_ is lower than that of MoSe_2_ QDs, suggesting a higher affinity of 2D CTAB-MoSe_2_ to H_2_O_2_ than MoSe_2_ QDs. Meanwhile, the lower *K*_m_ value of MoSe_2_ QDs to TMB represents its higher affinity in this respect. In addition, the parallel slope of the lines in the double-reciprocal plots of initial velocity versus different concentrations of one substrate reveals a ping-pong mechanism in the catalytic reaction [64,65,66]. This indicates that both of our MoSe_2_–based enzymes bound and reacted with the first substrate and the first product was subsequently released before the reaction with the second substrate.

### 3.5. Actives Species Trapping Tests and Peroxidase-Like Catalytic Mechanism

**Figure 11:**
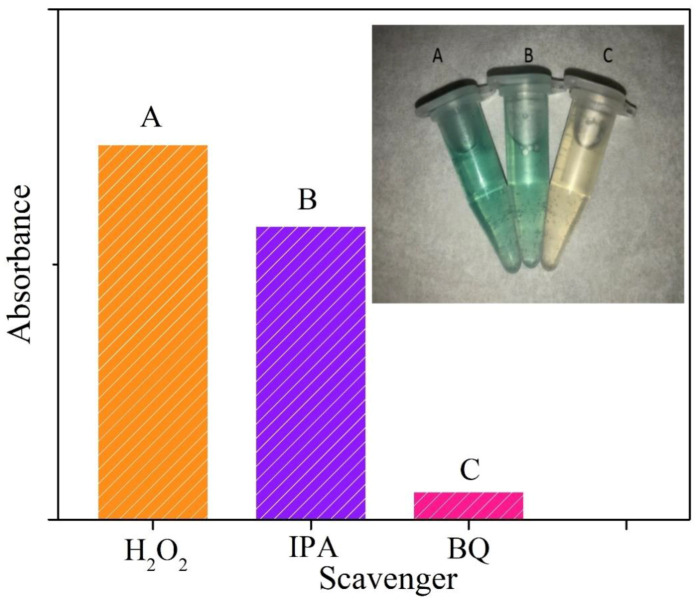
The absorbance at 652 nm of reaction solutions in the absence or presence of scavengers Isopropyl alcohol (IPA) and benzoquinone (BQ). The inset shows the images of color changes for different reaction systems.

**Figure 12:**
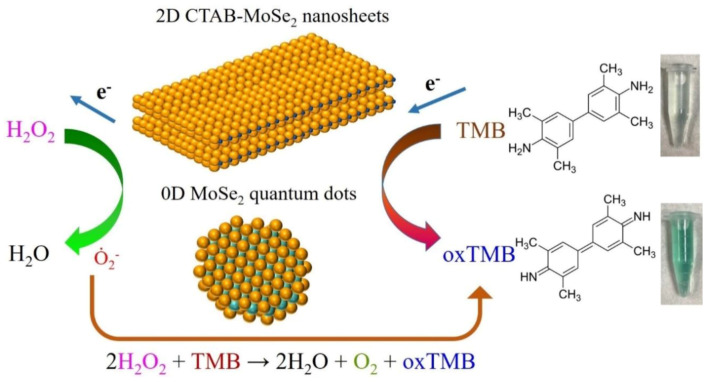
The schematic diagram depicts the mechanism for colorimetric detection of H_2_O_2_ by using 2D CTAB-MoSe_2_ NSs and MoSe_2_ QDs as peroxidase mimetics.

To confirm the prime species responsible for the peroxidase-mimetic catalytic activities of our artificial MoSe_2_–based enzymes, scavenger tests were employed. It is known that reaction systems involving hydrogen peroxide usually abound with reactive radicals such as ȮH and Ȯ_2_^−^. Then IPA and benzoquinone (BQ) were taken to be the scavengers in the reaction system for ȮH and Ȯ_2_^−^ radicals, respectively. Figure 11 shows the results of active species trapping tests of our reaction system. We found that the suppression of characteristic absorption and color fading were not evident with the addition of IPA. On the other hand, significant decrease in absorption and color contrast can be seen when BQ was added. This indicates that Ȯ_2_^−^ radical plays the major role to oxidize TMB to produce a TMB oxide and generate color contrast. Based on our finding and several previous reports [67,68], we propose the peroxidase-like catalytic mechanism of our MoSe_2_–based enzymes, which is illustrated in Figure 12. In the reaction process, TMB molecules are absorbed on the surface of MoSe_2_–based nanomaterials and act as the chromogenic electron donors. These molecules transfer their lone-pair electrons to MoSe_2_ from the amino groups, leading to the enhancement of electron density and mobility on the surface of MoSe_2_-based catalyst. In turn, it accelerates the electron migration from MoSe_2_–based catalyst to hydrogen peroxide. The one-electron transfer reaction generate a large amount of Ȯ_2_^−^ radicals that oxidize TMB and form blue-green product. Briefly, the MoSe_2_-based catalyst promote the electron transfer from TMB to H_2_O_2_, resulting in the oxidation of TMB and reduction of hydrogen peroxide. The production of colored oxTMB and water in this system can be expressed by the equation H_2_O_2_ + TMB → 2H_2_O + O_2_ + oxTMB.

### 3.6. Colorimetric Detection of H_2_O_2_ by MoSe_2_-Based Assay System

**Figure 13:**
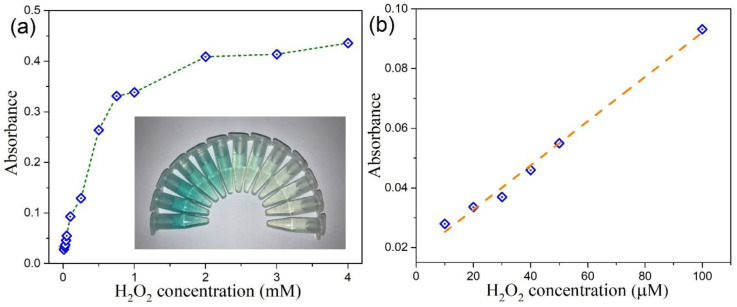
(**a**) The absorbance changes at 652 nm for MoSe_2_ QDs in different amount of H_2_O_2_ (0.01, 0.02, 0.03, 0.04, 0.05, 0.1, 0.25, 0.5, 0.75, 1, 2, 3 and 4 mM). Inset: the images of color contrast for different concentrations of H_2_O_2_. (**b**) The linear calibration plot for H_2_O_2_.

**Figure 14:**
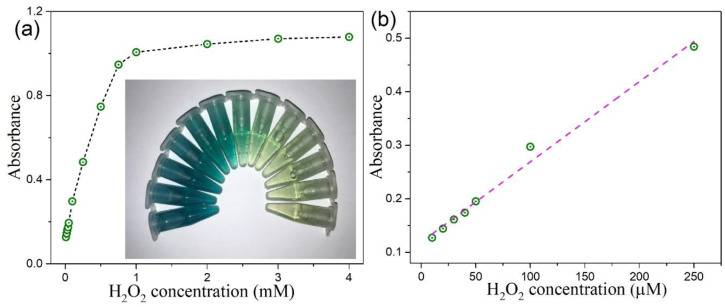
(**a**) The dose-response curve for H_2_O_2_ detection by using 2D CTAB-MoSe_2_ NSs as artificial enzyme. The inset are the photos of reaction solutions after adding different concentrations of H_2_O_2_. (**b**) The linear calibration plot for H_2_O_2_ concentration.

In view of the intrinsic peroxidase-like property of as-prepared MoSe_2_–based catalysts, a colorimetric strategy for the detection of H_2_O_2_ was established. The absorption spectra of TMB–H_2_O_2_–MoSe_2_ QDs system with different H_2_O_2_ concentration is presented in Figure S1. It can be seen that the characteristic absorption of TMB at 652 nm is dependent on the concentration of H_2_O_2_ varied from 10 μM to 4 M. Analogous results can be found with our TMB–H_2_O_2_–2D CTAB-MoSe_2_ system, as shown in Figure S2. Figure 13a and Figure 14a display the absorbance variations at 652 nm of the oxidized TMB in the presence of H_2_O_2_ with different concentrations for the TMB–H_2_O_2_–MoSe_2_ QDs system and TMB–H_2_O_2_–2D CTAB-MoSe_2_ system, respectively. Besides, the corresponding image in response to the change of H_2_O_2_ is shown in the insets of Figure 13a and Figure 14a, which shows the color variation could be seen by the naked eye. For TMB–H_2_O_2_–MoSe_2_ QDs system, the H_2_O_2_ concentration-response curve has a linear relationship in the range of 10 μM to 100 μM with a detection limit of 4 μM, as illustrated in Figure 13b. Figure 14b draws the calibration curve for H_2_O_2_ with a linear range from 10 to 250 μM for the TMB–H_2_O_2_–2D CTAB-MoSe_2_ system. The detection limit was also reckoned to be around 4 μM. It shows that the TMB–H_2_O_2_–2D CTAB-MoSe_2_ system could have a wider linear range compared with that of the TMB–H_2_O_2_–MoSe_2_ QDs system. Finally, we compare some representative colorimetric detections of H_2_O_2_ by using novel 2D materials in Table 1 [69,70,71,72]. It can be seen that the colorimetric sensing ability of H_2_O_2_ based on the peroxidase-like property of our MoSe_2_-based catalyst is comparable to other reported 2D materials-based colorimetric platforms. Therefore, our work provides a facile, simple, cost-effective, and alternative 2D materials-based colorimetric sensing platform for sensitive detection of H_2_O_2_. In principle, this sensing platform can be applied to a few diverse applications, yet proper selectivity tests should then be imposed to understand the specific accuracy in the measurement [31,73,74,75].

## 4. Conclusions

In summary, we prepared 2D and 0D MoSe_2_ nanostructures based on systematic and non-toxic top-down strategies. The characteristic excitation-dependent PL of the MoSe_2_ QDs can be attributed to the polydispersity of the synthesized QDs. The Raman shift of ultrathin MoSe_2_ nanosheets manifests the 2D nature of its structure. We demonstrated that these MoSe_2_ nanostructures possess intrinsic peroxidase-like activity in that they can facilitate the oxidation of TMB in the presence of H_2_O_2_, generating a visible color reaction. For the catalysis mechanism, kinetic analysis indicates that the catalytic reaction follows the typical Michaelis–Menten theory and a ping–pong mechanism. Moreover, active species study shows that Ȯ_2_^−^ plays a pivotal role in the peroxidase-like catalytic reaction. Based on the color reaction of TMB catalyzed by our MoSe_2_ nanomaterials, we have developed a new colorimetric method for H_2_O_2_ detection by using 2D and 0D MoSe_2_ nanostructures as peroxidase mimetics. It is shown that the colorimetric sensing capability of our MoSe_2_ catalysts is comparable to other 2D materials-based colorimetric platforms. Overall, the synthesis strategy we proposed is environmentally friendly and economic, and it can easily be adapted to construct novel inorganic low-dimensional enzyme-free mimetic with intrinsic catalytic activity. The potential of the presented 2D and 0D MoSe_2_ nanostructures for use as a catalyst in other oxidation reactions could be explored in the extended study and this could create a new opportunity for this enzyme-mimicking MoSe_2_ nanostructures in many significant fields, such as environmental protection, food monitoring, medical diagnostics, and photocatalysis.

## Figures and Tables

**Table 1:** Comparison of colorimetric detections of H_2_O_2_ in the linear range and detection limit between our MoSe_2_ nanostructures and other peroxidase mimics based on nanoscale 2D materials.

Catalyst	Linear Range (μM)	Detection Limit (μM)	Ref.
Positively-charged Au nanoparticles (NPs)	2–200	0.5	[15]
h-BN/N-MoS_2_	1–1000	0.4	[69]
Few-layered MoSe_2_ nanosheets (NSs)	10–160	0.4	[27]
MoS_2_ NPs	3–120	1.25	[23]
SDS–MoS_2_ NPs	2–100	0.32	[24]
g-C_3_N_4_	5–100	1	[70]
MoS_2_ QDs/g-C_3_N_4_ NSs	2–50	0.155	[71]
WS_2_ Nanosheets	5–200	1.5	[72]
2D CTAB-MoSe_2_	10–250	4	This work
0D MoSe_2_ QDs quantum dots (QDs)	10–100	4	This work

## Supplementary Materials

The following are available online at https://www.mdpi.com/2079-4991/10/10/2045/s1, Figure S1: UV–vis absorption spectra of MoSe_2_ QDs with different concentrations of H_2_O_2_, Figure S2: UV–vis absorption spectra of 2D CTAB-MoSe_2_ NSs with different amounts of H_2_O_2_, Table S1: Michaelis–Menten parameters of the 2D CTAB-MoSe_2_ NSs and 0D MoSe_2_ QDs.
