# Supplementary material for: 2D CTAB-MoSe2 Nanosheets and 0D MoSe2 Quantum Dots: Facile Top-Down Preparations and Their Peroxidase-Like Catalytic Activity for Colorimetric Detection of Hydrogen Peroxide
Source: Nanomaterials (Basel). 2020 Oct 16;10(10):2045. doi: 10.3390/nano10102045 (PMC7602750; doi:10.3390/nano10102045)
Supplement: Supplementary file 1 [file nanomaterials-10-02045-s001.pdf]

nanomaterials

Supplementary Material

2D CTAB-$MoSe_2$ nanosheets and 0D $MoSe_2$ quantum dots: Facile top-down preparations and their peroxidase-like catalytic activity for colorimetric detection of hydrogen peroxide

Da-Ren Hang [1,2,†,*], Ya-Qi Pan [1,†], Krishna Hari Sharma [1], Mitch M. C. Chou [1,2], Sk Emdadul Islam [3], Hui-Fen Wu [4] and Chi-Te Liang [3,*]

1 Department of Materials and Optoelectronic Science, National Sun Yat-sen University, Kaohsiung 80424, Taiwan; drhang@faculty.nsysu.edu.tw (D.-R.H.); zxcasd010379@gmail.com (Y.-Q.P.); krishnaharisharma.27@gmail.com (K.H.S.); mitch@faculty.nsysu.edu.tw (M.M.C.C.)
2 Center of Crystal Research, National Sun Yat-sen University, Kaohsiung 80424, Taiwan
3 Department of Physics, National Taiwan University, Taipei 10617, Taiwan; sk.emdadul87@gmail.com (S.E.I.); ctliang@phys.ntu.edu.tw (C.-T.L.)
4 Department of Chemistry, National Sun Yat-sen University, Kaohsiung 80424, Taiwan; hwu@faculty.nsysu.edu.tw

* Correspondence: drhang@faculty.nsysu.edu.tw; Tel.: +886-7-5252000 (ext. #4066) (D.-R.H.); ctliang@phys.ntu.edu.tw; Tel.: +886-2-33665129 (C.-T.L.)

† These authors contributed equally to this work.

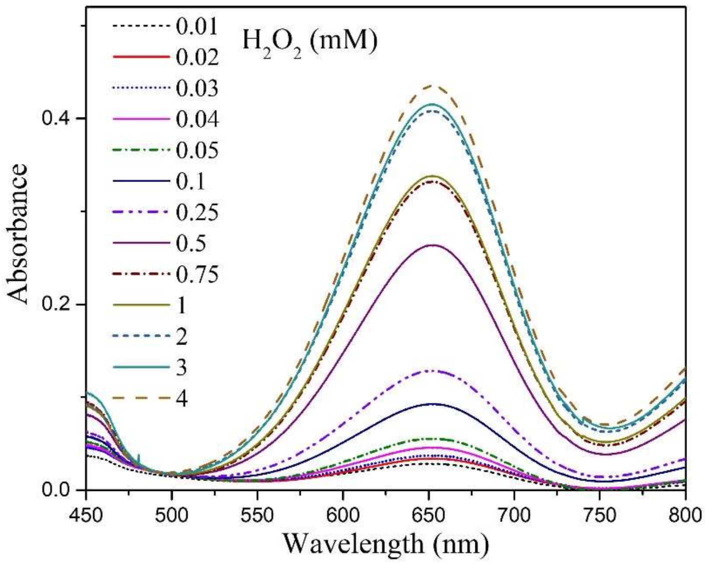

Figure S1. UV–vis absorption spectra of $MoSe_2$ QDs with different concentrations of H_2O_2.

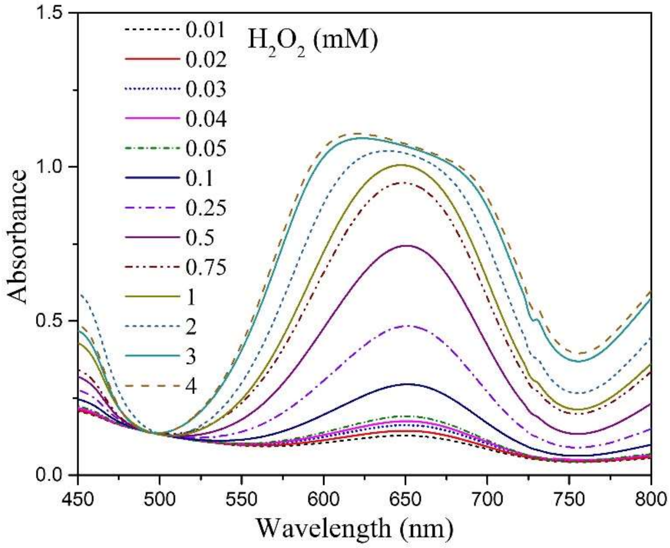

Figure S2. UV–vis absorption spectra of 2D CTAB-$MoSe_2$ NSs with different amounts of H_2O_2.

Table S1. Michaelis–Menten parameters of the 2D CTAB-$MoSe_2$ NSs and 0D $MoSe_2$ QDs.

Catalyst	Substrate	K_m (mM)	V_{max} (10^{-8} M s^{-1})
2D CTAB-$MoSe_2$	H_2O_2	0.015	0.03
nanosheets	TMB	4.22	0.076
0D $MoSe_2$ QDs	H_2O_2	0.389	0.012
	TMB	2.58	0.01

© 2020 by the authors. Submitted for possible open access publication under the terms and conditions of the Creative Commons Attribution (CC BY) license (http://creativecommons.org/licenses/by/4.0/).
